# Effects of Splenectomy on Spontaneously Chronic Pancreatitis in *aly/aly* Mice

**DOI:** 10.1155/2010/614890

**Published:** 2010-03-30

**Authors:** Heng-Xiao Wang, Shuang-Qin Yi, Jun Li, Hayato Terayama, Munekazu Naito, Shuichi Hirai, Ning Qu, Nozomi Yi, Masahiro Itoh

**Affiliations:** ^1^Department of Anatomy, Tokyo Medical University, 6-1-1 Shinjuku, Shinjuku-ku, Tokyo 160-8402, Japan; ^2^Department of Anatomy and Neuroembryology, University of Kanazawa, Takara-machi 13-1, Kanazawa 920-8640, Japan

## Abstract

*Background and Aim*. Mice with *alymphoplasia* (*aly/aly*) mutation characterized by a lack of lymph nodes, Peyer's patches, and well-defined lymphoid follicles in the spleen were found. In this study, we used splenectomized *aly/aly* mice to elucidate the effects of secondary lymphoid organs in the development of *aly/aly* autoimmune pancreatitis. *Methods*. Forty-eight 10-week-old *aly/aly* mice were divided into two groups for splenectomy and sham operation. Histological and immunohistochemical analyses of the pancreas were performed at the ages of 20, 30, and 40 weeks old after operation, respectively. *Results*. Our results showed that mononuclear cell infiltration was restricted to the interlobular connective tissues at the age of 20 weeks, and not increase obviously at the age of 30 and 40 weeks in splenectomized *aly/aly* mice. Furthermore, an apparent decrease in the expressions of CD4^+^ T, CD8^+^ T, and B cells was detected in the pancreatic tissues compared with sham *aly/aly* mice, however, no significant difference in macrophage expression between mice with and without a splenectomy. *Conclusions*. Inflammation infiltration and development of the pancreatitis in *aly/aly* mice were suppressed effectively after splenectomy, which was, at least partly, correlated to inhibition of the infiltration of T and B cells in pancreatic tissues but not to macrophages.

## 1. Introduction

Alymphoplasia/alymphoplasia (*aly/aly*) mice are autosomal recessive mutants of C57BL/6J (H-2^b^) strain possessing a point mutation in the gene-encoding NF-*κ*B-inducing kinase (NIK), which is characterized by a complete lack of lymph nodes and Peyer's patches [1–3], and may serve as a unique spontaneous model of Sjogren's syndrome [2]. The spleen of *aly/aly* mutants is devoid of well-defined lymphoid follicles and its white pulp is atrophic, and the thymus does not show clear cortical-medullary demarcation [1, 4]. Heterozygous *aly/+* mice, however, have a normal immune system and secondary lymphoid organs. The role of secondary lymphoid organs in immune responses has recently been evaluated using splenectomized *aly/aly* mice, which are considered to be completely lacking in secondary lymphoid organs [5, 6]. Lakkis et al. [7] and Yamanokuchi et al. [8] confirmed the necessity of secondary lymphoid organs in the initiation of allograft rejection, reporting that vascularized allografts were immunologically ignored by recipients in the absence of secondary lymphoid organs. Splenocytes isolated from *aly/aly* mice proliferate in response to both T and B cell mitogens and allogenic lymphocytes [1], and transferring these cells to recipient mice could lead mononuclear cells to infiltrate the pancreas [2]. The immunosuppressant agent FK506 can suppress the development of pancreatitis by enhancing apoptosis of the spleen T cells in *aly/aly* mice [9]. 

In our previous research, we performed sequential histopathology of pancreatic tissues in *aly/aly *mice. Our results showed that the pancreatic pathological signs of *aly/aly* mice start as early as 3 weeks of age, and that various populations of leukocytes are involved in the inflammation in *aly/aly* mice [10]. In this study, we used splenectomized *aly/aly* mice to elucidate the effects of secondary lymphoid organs in the development of *aly/aly* autoimmune pancreatitis. Furthermore, we investigated the effects of splenectomy on the infiltration of CD4^+^ T, CD8^+^ T, B cells and macrophages into pancreatic tissues and the relationship between mononuclear cells and pancreatitis developing in splenectomized *aly/aly* mice, which have a complete absence of secondary lymphoid organs.

## 2. Materials and Methods

### 2.1. Animals

Male *aly/aly *and female *aly/+ *mice (8 weeks old) were purchased from Nihon Clea (Japan) and were housed under control illumination (12:12 light/dark cycle, lights on at 8:00 a.m.) and temperature 25 ± 2°C, with standard chow F-1 (Nippon Funabashi Farm Co., Ltd. Japan). The metabolizable energy content of the pellets was 376 kcal/100 g, and the pellets consisted of 21.3% protein, 51% fat, 3.1% fiber, 5.0% ash, and 57.5% complex carbohydrate. The feed and water were supplied ad libitum. Since the adult *aly/aly *female cannot suckle neonates due to an imparted mammary gland development, the strain was bred by intercrossing heterozygous females with homozygous males [1]. The offspring were weaned from their mothers at 4 weeks and identified as *aly/aly* or *aly/+* mice by measuring serum IgA levels at the age of 6 weeks. All animals were bred and maintained in our animal facility in Tokyo Medical University under pathogen-free conditions, and treated in accordance with the guide for animal experimentation of Tokyo Medical University.

### 2.2. Identification of aly/aly or aly/+ Mice

Blood samples were collected via the tail vein of offspring mice of 6 weeks, and centrifuged (1500 g, 15 minutes at 4°C). Serum samples were removed. IgA was measured using a commercially available ELISA quantitation kit (Code, E90-103-26; Bethyl Laboratories, Inc., Montgomery, TX 77356, USA) following the manufacturer's instructions. Briefly, the coating buffer diluted in capture antibody of goat anti-mouse IgA-affinity purified was added to 96-well culture plates, after 60-minute incubation at room temperature (RT), and then the capture antibody solution was aspirated from each well, and washed three times with washing buffer. Blocking solution was added to each well and incubated for 30 minutes at RT, after which the blocking solution was removed and washed three times. After the serum had been diluted in sample diluent buffer (1 : 5), the serum samples and standards were transferred to their assigned wells and, after 60 minutes incubation at RT, the samples and standards were removed and rinsed five times with washing buffer. The plates were incubated with conjugate diluent added to the HRP-conjugated anti-IgA antibody (Code, E101-115; Bethyl Laboratories, Inc.) for 30 minutes at RT, and the HRP conjugate was removed, and washed five times with washing buffer. Finally, the plates were incubated for 15 minutes at RT by substrate solution with shading. The reaction was stopped with 2 M H_2_SO_4_ solution. Absorbance at 450 nm was immediately measured using a plate reader (ImmunoMini NJ-2300; Tokyo, Japan). Nonspecific binding was determined using normal mouse IgA and subtracted from the experimental samples. ELISA ratios were calculated with Excel. The mice with inhibited serum levels of IgA 0.005–0.01 mg/dl were identified as homozygous *aly/aly* mice, while the serum levels of IgA >0.3 ± 0.03 mg/dl were identified as *aly/+ *[4]. For the results, mice exhibiting serum IgA levels of 0.004–0.005 mg/dl were identified as homozygous *aly/aly* mice, and of 0.33–0.35 mg/dl were identified as heterozygous *aly/+* one. About 40% of the offspring mice were identified as* aly/aly*, and no significant differences between male and female were detected in the identified homozygous *aly/aly* mice.

Furthermore, as our previous study, homozygous *aly/aly* mice were also confirmed by checking whether or not there were any mesenteric lymph nodes. Mice of nonmesenteric lymph nodes were identified as homozygous *aly/aly* mice [10].

### 2.3. Splenectomized

Ten-week-old homozygous* aly/aly *mice, with no sex limitation, were randomly divided into 2 groups, an operation group (*n* = 24) and a sham group (*n* = 24). For the operation group, splenectomy was performed on a clean bench using standard techniques, as previously detailed [11, 12]. Briefly, the *aly/aly* mice were first anesthetized with ether and then given an intraperitoneal injection of 10 mg/kg sodium pentobarbital solution (Somnopentyl; New Jersey, USA). After the animals were completely anesthetized, an approximately 1 cm skin incision was made in the skin of the left lumbar region, and the peritoneal membrane was opened to expose the spleen. The spleen was removed from the edge of the pancreatic tissues, and the splenic artery and vein were ligated at the hilum lienis, and then the spleen was slowly removed intact. During the operation, care was taken to avoid damaging the pancreas and surrounding tissues. This procedure took about 10–15 minutes, and it was ensured that the spleen had all been removed and that no splenic fragments were left behind, which was confirmed by examining the mice at the time of death. For the sham group, the *aly/aly* mice underwent a laparotomy only and, after five minutes, the peritoneal membrane and skin were then closed separately. Both mouse groups were returned to their environment before the operation, breeding until sacrificed.

### 2.4. Tissue Preparation

The mice in splenectomized and sham groups were sacrificed 10 weeks (*n* = 8), 20 weeks (*n* = 8), and 30 weeks (*n* = 8) after the operation, respectively. Briefly, after the mice had been completely anesthetized, the abdominal cavity was opened, and a catheter was inserted retrogradely into the abdominal aorta at a level immediately above the bifurcation of this artery into the common iliac arteries. Perfusion was commenced with 0.1 M phosphate buffer solution (PBS, pH 7.4) containing heparin (10 IU/mL) and thereafter with periodate-lysine-paraformaldehyde solution (PLP) (4% paraformaldehyde containing 0.075 M lysine and 0.01 M sodium periodate solution PH 7.4) [13]. Samples of the pancreas were harvested and fixed in PLP solution at 4°C for 5 h. They were then dehydrated in 30% sucrose solution (30 g sucrose and 100 mL 0.01 M phosphate buffer) at 4°C for 3 h two times. The samples were then embedded in OCT compound (Tissue-Tek; Mile, Elkhart, Ind., USA), and quickly frozen in liquid nitrogen. Sections (6 *μ*m) were cut and placed on gelatin-coated glass slides, and stored at −30°C until used.

### 2.5. Histology and Immunohistochemistry

The sections were stained with hematoxylin-eosin (HE) and then examined with light microscopy.

Immunostaining was performed according to our previous papers [10, 14]. Briefly, the sections were immersed in Block Ace (Dainippon Pharmaceutical Co., Japan) containing 0.3% (v/v) hydrogen peroxide for 10 minutes to block endogenous peroxidase activity. After rinsing in 0.01 M PBS, the sections were blocked with 1.5% normal rabbit serum for 20 minutes at RT to reduce nonspecific staining, and incubated with the primary antibodies for 2 h at RT in a humidified chamber, and then with the secondary antibodies for 30 minutes at RT. Subsequently, the avidin-biotin-complex technique (ABComplex/HRP; Vector Laboratories, Inc., Burlingame, CA, USA) was performed by incubating the sections with ABC complexes for 30 minutes at RT, and then treating them for 1 minute with 3-3-diaminobenzidine and 0.005% H_2_O_2_, used as chromogens. The sections were counterstained with Harris hematoxylin for 2–5 minutes, dehydrated in a graded ethanol series and xylene, and coverslipped with Entellan neu (Merck, Germany).

The primary antibodies were rat anti-mouse CD4 antibody (monoclonal, no. 550280; BD Pharmingen), rat anti-mouse CD8a(Ly-2) antibody (monoclonal, no. 550281; BD Pharmingen), rat anti-mouse CD45R/B220 antibody (monoclonal, no. 550286; BD Pharmingen), and rat anti-mouse F4/80 antibody (monoclonal, no. ab6640; Abcam), diluted at 1 : 40, 1 : 100, 1 : 200, and 1 : 1000, respectively. The secondary antibody was anti-rat IgG biotinylated antibody (AK 5004; Vectastain), diluted at 1 : 200.

The control experiments consisted of the following: (1) omission of primary antiserum and (2) substitution of primary antibody with 0.05 M Tris-BSA buffer. These controls were carried out on sections at the same time as the treatment with the primary antibody.

### 2.6. Histopathological Evaluation of Pancreatitis

The histopathological evaluation of pancreatic lesions was performed under light microscopy (OLYMPUS Bx51, Tokyo, Japan; objective ×400). The inflammatory lesions of the pancreas were grated according to Tsubata et al.'s arbitrary scoring system [2]. The number of positively stained cells was observed under a light microscopy. The counting was carried out in four to five different fields in at least one section of the pancreas by eight of splenectomized* aly/aly* mice and sham *aly/aly* mice, respectively.

### 2.7. Statistical Analysis

Differences between splenectomized and sham-splenectomized *aly/aly* mice were analyzed by Student's *t*-test, and *P* < .05 was considered significant.

## 3. Results

### 3.1. Histopathological Analysis

**Figure 1:**
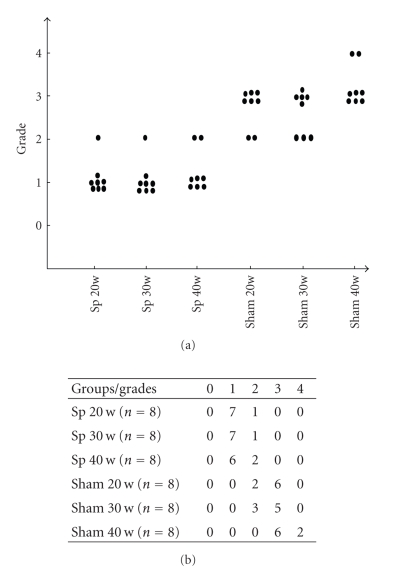
Chronic inflammatory changes of splenectomized (Sp) and sham *aly/aly* mice at the ages of 20, 30, and 40 weeks old, respectively. The pancreas was histopathologically examined (refer to Figure 2). Grading of inflammatory lesions of the pancreas: 0, no visible change; (1), focal accumulation of lymphoid cells; (2), focal accumulation of lymphoid cells with evidence of tissue damage; (3), extensive lymphoid cell infiltration with tissue damage (<30%); (4), replacement by adipose tissue (>30%).

System histopathological analysis was carried for 48 *aly/aly* mice of 20, 30, and 40 weeks old in either the splenectomized or sham groups. Figure 1 summarizes the chronic inflammatory changes of the pancreas in splenectomized and sham-group *aly/aly* mice. The inflammatory lesions of the pancreas were graded according to Tsubata et al.'s arbitrary scoring system [2]. The examination revealed the development of chronic inflammatory lesions in the pancreas of aged sham-group *aly/aly* mice, while in splenectomized-group mice it showed minimal mononuclear cell infiltration and no tissue destruction at the ages of 20, 30, and 40 weeks old (Figure 1).

**Figure 2:**
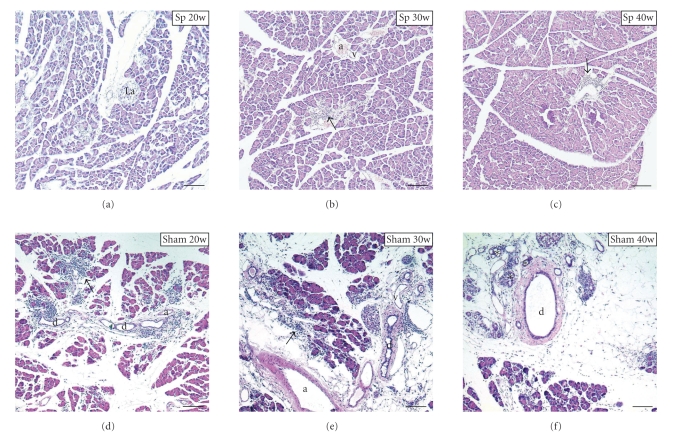
Histopathological analysis of effects of splenectomy (sp) on pancreatitis developing in *aly/aly* mice at the ages of 20, 30, and 40 weeks old, respectively. Sham 20 w, 30 w, and 40 w indicated sham-group mice at the ages of 20, 30, and 40 weeks old, respectively. Arrows, indicating lymphoid cell infiltration and *indicating some pancreatic ducts of proliferation and regeneration. a, artery; d, pancreatic duct; La, islet of Langerhans; v, vein. Scale bars: 200 *μ*m.

In splenectomized group mice, minimal mononuclear cells infiltrated the interlobular connective tissues around the vascular and lymphatic vessels of the pancreas at the age of 20 weeks. The acini cells were arranged properly around small lumen, and the intralobular and interlobular ducts did not show expansion (Figure 2(a)). Moreover, in 30- and 40-week-old splenectomized mice, minimal to medium mononuclear cell infiltration into the pancreas was confined only to local tissues of intralobular spaces or around the interlobular ducts, destroyed tissues or partial fatty changes were also not found, and the islets had not changed and remained intact (Figures 2(b) and 2(c)).

On the other hand, in 20-week-old sham-group *aly/aly* mice, inflammation cell infiltration occurred significantly in the pancreatic parenchyma and interlobular connective tissue around vessel and interlobular ducts. Typical lesions were characterized by lymphocyte accumulation in periductal areas and cell infiltration extending to the lobules (Figure 2(d)). In 30- and 40-week-old mice, the lymphoid cells began to infiltrate the pancreatic tissues from connective tissues throughout the disease progression. Marked lymphoid cell infiltration with destruction of acini and lobules, degeneration of most acinar cells adjacent to infiltrating cells, and partial fatty changes were observed (Figures 2(e) and 2(f)), and some islets in fatty changed areas seemed to become smaller in the pancreas of 40-week-old mice (Figure 2(f)).

### 3.2. Immunohistological Changes after Splenectomy

**Figure 3:**
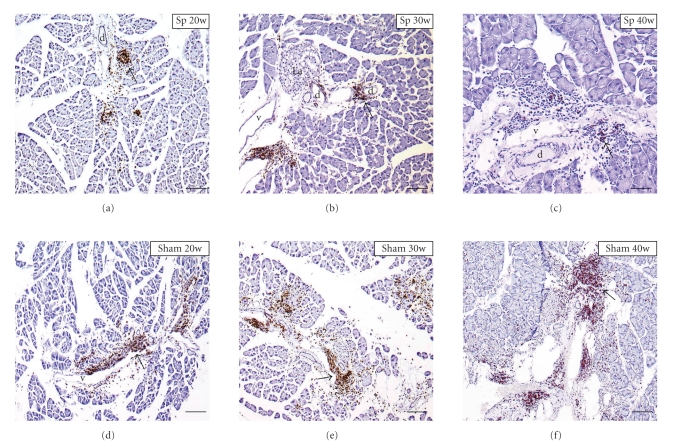
Immunohistopathological analysis of pancreas in splenectomized (Sp) and sham *aly/aly* mice by CD4. Arrows, indicating CD4-positive cells. a, artery; d, pancreatic duct; La, islet of Langerhans; v, vein. Scale bars: 100 *μ*m (c); 200 *μ*m ((a), (b), (d), (e), and (f)).

In 20-week-old splenectomized *aly/aly* mice, a slight expression of CD4+ cells in the basal layer and in regional interlobular connective tissues around the vessels and exocrine gland was recognized (Figure 3(a)). In 30-week-old operated mice, the expression of CD4+ cells was observed in similar pancreatic areas in 20-week-old mice (Figure 3(b)), and the numbers of CD4+ cells were not increased compared to 20-week-old mice (Figure 5). In 40-week-old operated *aly/aly* mice, CD4+ infiltrated cells were hardly seen (Figure 3(c)). 

However, in sham-group *aly/aly* mice, marked expression of CD4+ cells in interlobular connective tissues around the vessels was observed in the pancreas of 20-week-old mice (Figure 3(d)). In 30- and 40-week-old mice, CD4+ cells were extensively expressed in the pancreatic tissues around the blood vessels and interlobular ducts (Figures 3(e) and 3(f)). Overall, the number of CD4+ cells in splenectomized *aly/aly* mice fell significantly compared with sham *aly/aly* mice of the same age (Figure 5).

**Figure 4:**
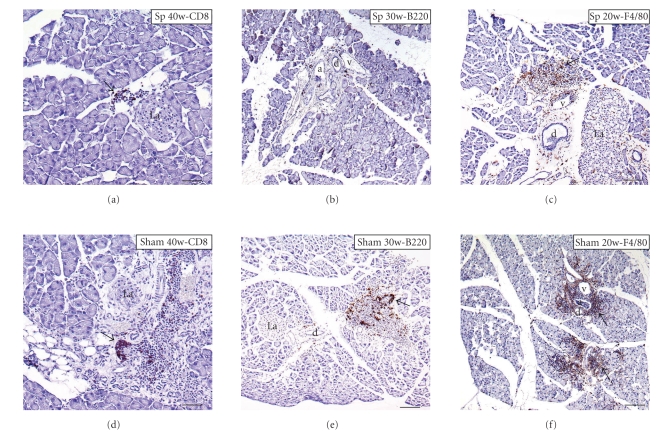
Immunohistopathological analysis of pancreas in splenectomized (Sp) and sham *aly/aly* mice by CD8a, B220, and F4/80. Arrows, indicating CD8- ((a) and (d)), B220- (e), and F4/80- ((c) and (f)) positive cells. a, artery; d, pancreatic duct; La, islet of Langerhans; v, vein. Scale bars: 100 *μ*m ((a), (b), and (d)); 200 *μ*m ((c), (e), and (f)).

**Figure 5:**
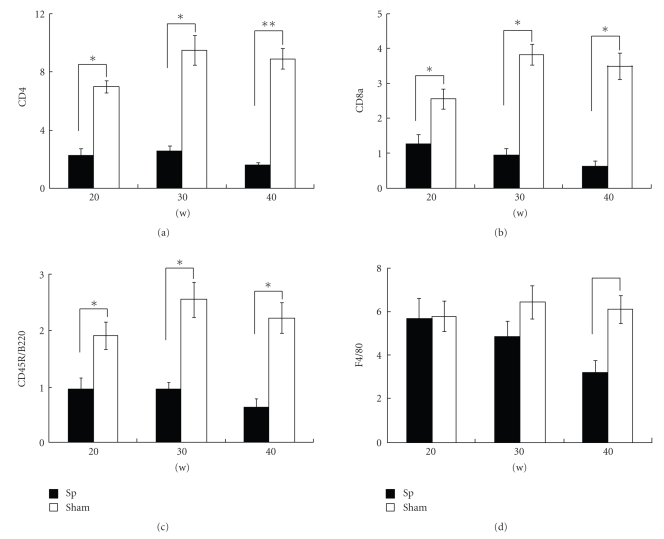
Immunostaining positive scoring of the pancreas was evaluated as a percentage of the total area of the specimen in splenectomized (Sp) and sham *aly/aly* mice at the ages of 20, 30, and 40 weeks old, respectively. Results are expressed as the mean ± SEM. *P < .05; **P < .01.

CD8a+ cell expression in the pancreas of 20-, 30- (data not shown), and 40-week-old mice was recognized around the regional interlobular ducts and vessels (Figure 4(a)). The number of CD8+ cells was about threefold reduced obviously in the pancreatic tissues of splenectomized-group mice compared with sham-group mice of the same age (Figures 4(d) and 5).

In splenectomized *aly/aly* mice, B220+ cells were minimal in the pancreatic tissues of 20- and 40-week-old mice (data not shown), and hardly seen in infiltrated pancreatic tissues of 30-week-old mice (Figure 4(b)); however, the expression of B220+ cells in splenectomized-group mice was significantly lower than in sham-group animals of the same age (Figure 5).

In 20-week-old *aly/aly* mice, F4/80+ cells filtrated the interlobular tissues of the pancreas in splenectomized- and sham-group animals (Figures 4(c) and 4(f)). Although the expressions of F4/80 in 30- and 40-week-old splenectomized *aly/aly* mice were reduced (Figure 5), this phenomenon was not continual in 45- and 50-week-old animals (data not shown). There was no statistically significant difference in the expression of F4/80+ cells in pancreatic tissues among the two groups (Figure 5).

In summary, we compared CD4, CD8a, CD45R/B220, and F4/80 distribution in the pancreatic tissues of splenectomized and sham *aly/aly* mice. In the experimental group, the expressions of CD4+, CD8a+, and CD45R/B220+ cells were only in regional pancreatic tissues of 20-week-old *aly/aly* mice, and positive cells were not increased in 30- and 40-week-old operated *aly/aly* mice. However, the expression of F4/80+ cells did not change in the pancreas between 20-week-old mice with and without a splenectomy, and did not significantly fall in 30- and 40-week-old splenectomized *aly/aly* mice.

## 4. Discussion

In this present study, the histological and immunohistochemical analysis of pancreatic tissues in splenectomized *aly/aly* mice was examined. Our results indicated minimal to medium mononuclear cell infiltration in pancreatic tissues of splenectomized *aly/aly* mice at the age of 20 weeks, and infiltration was restricted to the interlobular connective tissues around the vascular and lymphatic vessels. Moreover, the infiltrated mononuclear cells did not clearly increase and the destroyed tissues also did not appear in 30- and 40-week-old splenectomized *aly/aly* mice. Therefore, inflammation infiltration and development of pancreatitis were suppressed effectively after splenectomy in *aly/aly* mice.

The spleen, as the primary secondary lymphoid organ, provides an environment in which antigen is efficiently retained, presented, and ordered in cellular interactions between antigen-presenting cells (APCs), T cells, and B cells [15]. In *aly/aly* mice, the absence of lymphotoxin beta receptor signaling and NF-*κ*B-induced kinase leads to irreversible degeneration of lymph node stroma, furthermore leading to an absence of peripheral lymph nodes and Peyer's patches [16, 17], but the spleen remains in adult *aly/aly* mice despite a lack of well-defined lymphoid follicles. An abnormal splenic microarchitecture may contribute to the immune deficiencies described in *aly/aly* mice [18], and the splenocytes of *aly/aly *mice can lead to pancreatitis by transferring to recipient mice [2]. In this present study, surgical resection of the spleen in 10-week-old *aly/aly* mice showed that inflammation cells infiltrating pancreatic tissues were markedly decreased at the ages of 20, 30, and 40 weeks old compared to *aly/aly* mice without splenectomy. This indicated that a splenectomy can suppress inflammation cells infiltrating the pancreas in *aly/aly* mice. Furthermore, it was suggested that lymphocytes infiltrating the pancreas mainly arose from the spleen in *aly/aly* mice.

In this study, an apparent decrease in the expressions of CD4^+^ T, CD8^+^ T, and B cells was detected in the pancreatic tissues of 20-week-old *aly/aly* mice which were splenectomized at the age of 10 weeks old compared with 20-week-old sham *aly/aly* mice. Furthermore, the infiltration of CD4^+^, CD8^+^ T cells, and B cells did not increase in the pancreas of 30- and 40-week-old splenectomized *aly/aly* mice. This implied that treatment with splenectomy in *aly/aly* mice apparently decreased the infiltration of T cells and B cells into the pancreas, and interrupted pancreatitis development. 

The study in splenectomized patients and mice demonstrated that the function of the spleen is mainly to filter particulate and soluble antigens from the blood. Dai and Lakkis demonstrated that the number of mature naive CD4^+^ T cells in the blood of adult thymectomy splenectomized *aly/aly* mice was significantly lower than that of thymectomized mice [6]. Splenectomized mice showed a remarkably decreased amount of viable bacteria in the liver than the sham mice after *Listeria *challenge [19]. Furthermore, most of the intestinal intraepithelial T lymphocyte subpopulations in *aly/aly* mice develop independently of passage through Peyer's patches and mesenteric lymph nodes [20]. Intestinal intraepithelial T lymphocytes would be a source of infiltrated cells in nonlymphoid tissues in splenectomized *aly/aly* mice. Moreover, B lymphocytes in the bone marrow of splenectomized mice were found to be fully immunocompetent, and this compensatory mechanism proved to result in an almost normal function of the humoral immune system in splenectomized mice [21], and the peritoneal cavity contained more B1 cells in *aly/aly* mice than in normal mice [22]. With that, in splenectomized *aly/aly* mice, although the number of T cells and B cells infiltrating the pancreas apparently decreased, because of the influence of intestinal intraepithelial T cells, and immunocompetent B-cell function, positive T and B cells were also found in pancreatic tissues.

Additionally, our previous studies showed that many macrophages and eosinophilic granulocytes first appeared and remained in pancreatic lymphatic lumina and the interlobular connective tissues in the early stage in *aly/aly* mice [10]. In the present study, many macrophages infiltrating the interlobular connective tissues were noted in the pancreas of 20-week-old sham *aly/al*y mice. At one time, numerous macrophages were also found in the same areas of 20-week-old splenectomized *aly/aly* mice. These phenomena were also found at the ages of 30 and 40 weeks old in either splenectomized or sham mice, and no statistically significant difference in the expression of macrophages in pancreatic tissues was found between mice with and without a splenectomy. It might be considered that macrophages had infiltrated the pancreas in the early stage in splenectomized *aly/aly* mice. Moreover, activated macrophages display foreign substances in a form that can be recognized and responded to by lymphocytes [23]. This may be another reason why infiltrated macrophages could not be affected in pancreatic tissues by splenectomy in *aly/aly* mice. 

In conclusion, the present study demonstrated for the first time that a splenectomy can suppress inflammation cells from infiltrating the pancreas in *aly/aly* mice. Therefore, it was suggested that lymphocytes infiltrating the pancreas mainly come from the spleen in *aly/aly* mice. Furthermore, our results implied that it was, at least partly, correlated to inhibition of the infiltration of  T and B cells in pancreatic tissues but not to macrophages. Removal of the spleen is usually well tolerated and inhibits lymphocyte activation, but cannot fully control the infiltration of lymphocytes into the pancreas in *aly/aly* mice. Since other leukocyte subsets and various cytokines, such as dendritic cells [24], IL-2, and GM-CSF [25], also affected the pancreatitis development in *aly/aly* mice, it will be interesting to examine the connection between splenectomy and these factors in the further study.
